# Training, executive, attention and motor skills (TEAMS) training versus standard treatment for preschool children with attention deficit hyperactivity disorder: a randomised clinical trial

**DOI:** 10.1186/s13104-018-3478-3

**Published:** 2018-06-08

**Authors:** Helle Annette Vibholm, Jesper Pedersen, Erlend Faltinsen, Michael H. Marcussen, Christian Gluud, Ole Jakob Storebø

**Affiliations:** 1 Child and Adolescent Psychiatric Department, Region Zealand, Roskilde, Denmark; 2 Psychiatric Research Unit, Region Zealand Psychiatry, Slagelse, Denmark; 3 Copenhagen Trial Unit, Centre for Clinical Intervention Research, Rigshospitalet, Copenhagen University Hospital, Copenhagen, Denmark; 4 Department of Psychology, University of Southern Denmark, Odense, Denmark

**Keywords:** Attention deficit hyperactivity disorder, Randomised clinical trial, Behavioural intervention, Children, Adolescents

## Abstract

**Objective:**

This study compared the effectiveness of manualised training, executive, attention, and motor skills (TEAMS) training versus standard treatment in preschool children with attention deficit hyperactivity disorder (ADHD). We conducted a randomised parallel group, single-blinded, superiority trial. The primary outcome was ADHD symptoms and the secondary outcome was functionality. Parents and primary school teachers assessed outcomes at pretreatment, posttreatment, and at one, three, and 6 months follow-up.

**Results:**

In total, 67 children (aged 3–6 years) were randomised. In the TEAMS group, 32 out of 33 (97%) participants completed the total 8-week program, compared with only 7 out of 26 (27%) in the control group. The repeated-model analyses showed no significant change between the two interventions for ADHD symptoms and functionality levels over time. The mean difference in ADHD symptoms between TEAMS versus standard treatment at posttreatment was 2.18 points (95% confidence interval − 8.62 to 13.0; trial sequential analysis-adjusted confidence interval − 19.3 to 23.7).

*Trial registration* Clinical Trials identifier: NCT01918436 (Retrospectively registered). Registered on 7 August 2013.

**Electronic supplementary material:**

The online version of this article (10.1186/s13104-018-3478-3) contains supplementary material, which is available to authorized users.

## Introduction

Attention deficit hyperactivity disorder (ADHD) is a prevalent and chronic neuropsychiatric disorder with three core symptoms: inattention, hyperactivity, and impulsivity [1]. Neuroimaging and neuropsychological data have indicated growth and functional anomalies throughout the neocortex, white matter pathology, and disrupted anatomical connectivity in the brains of children with ADHD [2]. ADHD also affects neurocognitive functions and behavioural processes, including working memory, planning, and inhibitory control [2, 3].

Early childhood is a particularly vital and responsive period in brain development [4, 5]. Interventions that target neural growth and development may enable more sustainable ADHD treatments [6]. However, the long-term benefits and adverse events of pharmacological and non-pharmacological treatments need to be thoroughly investigated [7–9]. The training, executive, attention, and motor skills (TEAMS) program is a non-pharmacological and neurocognitive training program that targets preschool children with ADHD [10, 11]. Preliminary data from Halperin and colleagues have been favourable [10, 11], but randomised clinical trials on TEAMS training are lacking. We aimed to assess whether TEAMS training could significantly improve ADHD symptoms and functionality levels in Danish preschool children with ADHD, compared with standard treatment.

## Main text

### Methods

#### Trial design

We conducted a randomised parallel group, single-blinded, superiority trial. A comprehensive description of the trial design and rationale has been published elsewhere [12]. The study protocol was registered on ClinicalTrials.gov with the ID number NCT01918436. The trial obtained approval from Region Zealand’s Committee on Health Research Ethics in Denmark, with the project number SJ-331 and the registration number 34758. Informed, written, and signed consent was obtained by the participating children’s legal guardians. All participants were free to leave the trial, and if a child psychiatrist or legal guardian determined that a child needed pharmacological treatment, this was endorsed and the child was excluded. Any adverse events were reported. The trial adhered to the CONSORT checklist.

#### Participants

The recruitment period lasted from January 1, 2012 to October 31, 2015. Eligible children were referred from four child and adolescent psychiatric clinics in Region Zealand, Denmark, to an ADHD outpatient clinic in Holbaek, Denmark, where randomisation and both interventions were carried out. The interventions lasted for 8 weeks, with follow-up assessments at 1-, 3-, and 6-months.

Participants were included if they: (a) had a formal diagnosis of ADHD according to the diagnostic and statistical manual of mental disorders [1]; (b) were 3–6 years of age at baseline; and (c) their parents were willing to participate in the trial and consented to the children’s participation. Children were excluded if they: (a) lived in a residential institution or in an unstable environment outside the home; (b) did not speak or understand Danish; (c) were taking ADHD medication; (d) had significant disabilities as a result of physical or psychiatric comorbidity; or (e) had parents who were not capable or willing to cooperate in implementing the program.

#### Randomisation, treatment allocation, and blinding

The randomisation was conducted with the web-based tool OPEN Randomize [13]. We used central randomisation with computer generated, permuted randomisation sequences in blocs of four, and an allocation ratio of 1:1, stratified for sex and age. Participants, parents, treating physicians, or health personnel were not blinded to treatment allocations. Outcome assessors and the statistician were blinded to allocations [12].

#### Measures

Parents and teachers rated the primary outcomes at baseline, at post-treatment, and at follow-ups. The ADHD rating scale-IV (ADHD-RS IV) (Danish child version, ages 5–10 years) is a norm-referenced checklist, and contains 26 items that measure ADHD symptomology, including hyperactivity/impulsivity, inattention, and conduct problems [14, 15].

The strengths and difficulties questionnaire—Denmark (SDQ-DAN) is a behavioural screening questionnaire for children between 2 and 17 years, and measures emotional, conduct, hyperactivity, and peer problems [16]. Please see the protocol for a description of their psychometric properties [12].

#### Treatment groups

The participants in the TEAMS intervention were divided into groups of two to five families, who had 8 weekly 45-min meetings with separate parent and child sessions. The parent groups mainly consisted of psychoeducation and collaborative problem solving [12]. The children groups consisted of problem solving, plus aerobic exercise, and relaxation techniques.

The control group was the conventional treatment regime for preschool ADHD patients, as outlined by the Danish national clinical guidelines [17]. It entailed a combination of psychoeducation, socialising, and cooperation activities. For a comprehensive description of both interventions, please see the protocol [12].

#### Data analysis

The sample size was calculated based on a type I error (α) of 5% and a type II error (β) of 20% (i.e. power of 80%), with an allocation ratio of 1:1 [12]. We used a standard deviation of five points. With an estimated withdrawal of approximately 30 patients, we planned to randomise a total of 120 participants [12]. The analyses were primarily conducted with and without adjustment for stratification variables. SPSS Statistics 22 was used to conduct the analysis.

The significance level was set at p < 0.05. Prior to each analysis all effect measures were inspected for normality (test of kurtosis and skewness, as well as a Shapiro Wilks test, plus inspection of histograms and distributions).

Descriptive statistics were generated. We used a stepwise procedure to test the effect of the intervention and the effect of time. The effect size was estimated by mean differences and confidence intervals (CI) at baseline, posttreatment, 1, 3, and 6 months using independent sample T tests. Subsequently, we performed adjusted analyses with an analysis of covariance model (ANCOVA) to incorporate covariates, pre-score (for ADHD-RS-IV and SDQ-DAN, respectively), and group as a fixed-factor. We compared the difference between baseline and 8 weeks after the TEAMS intervention program, as well as the difference between the two groups. The ANCOVA model allowed us to look for differences in adjusted means (i.e. adjusted for the covariates). Finally, we measured the effects of the TEAMS intervention at different time points and between groups by using a general linear repeated measure model (GLM). In advance, model fit was assessed for each regression model.

As we were unable to reach the planned sample size, we conducted a post hoc trial sequential analysis on the primary teacher-rated outcome ADHD-RS IV at posttreatment. Trial sequential analysis is a tool for quantifying the statistical reliability of data by adjusting significance levels for sparse data and repetitive testing for accumulating data [18–23]. The required sample size was calculated based on a minimal relevant difference of 3 points, a standard deviation of 5 points, an alpha of 3.33% (to take into consideration that we had two primary outcomes), and a beta of 10%.

### Results

**Fig. 1:**
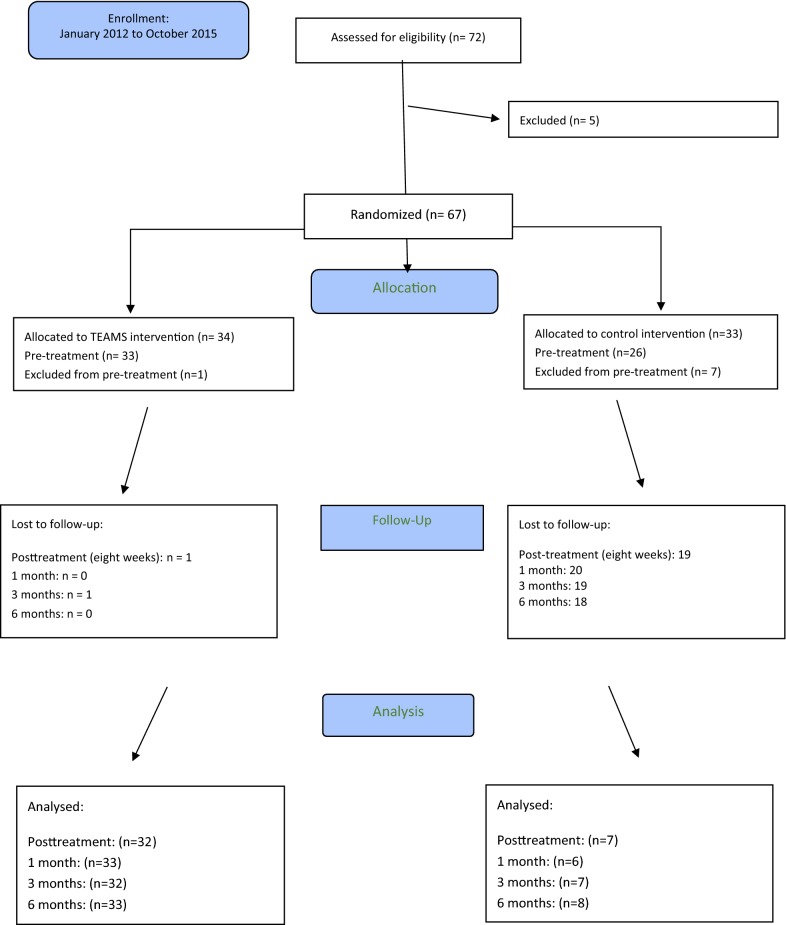
Flow chart of group allocations

Only 72 participants were eligible during the trial inclusion phase from January 1, 2012 to October 31, 2015, and five declined to attend the trial. This left us with 67 eligible participants to be randomised (Fig. 1). 65 children were of Danish ethnicity, whereas one boy and one girl had non-Danish ethnicities. 34 children (32 boys, 2 girls) were randomised to the TEAMS experimental group, and 33 children (31 boys, 2 girls) to the control group. One participant dropped out in the TEAMS intervention prior to baseline assessment, and seven participants in the control intervention (Fig. 1).

In the TEAMS group, 32 out of 33 (97%) participants completed the total 8-week program. 33 were assessed at 1-month follow-up, 32 at 3 month follow-up and 33 and 6 months follow-up. Seven participants (21%) from the control group completed the total 8-week program. In turn, 20 (77%) participants were lost at 1-month follow-up, 19 (73%) at 3 months follow-up, and 18 (69%) at 6 months follow-up. Because of the substantial attrition rates in the control group, the intention-to-treat principle and multiple imputations were not conducted. Age, sex, and baseline ADHD-RS-IV and SDQ-DAN scores were balanced between the groups (Additional file 1).

ADHD symptoms changed positively over time (Table 1). The ANCOVA model showed no significant difference between the two groups for both scores (ADHD-RS-IV and SDQ-DAN) when we adjusted for baseline values and group (Additional file 2). The repeated measures GLM procedure illustrates the effect over time and between groups: results showed that ADHD symptoms changed significantly over time (p = 0.020) and no significant change appeared in the SDQ scores (p = 0.166). Over time and between groups, results were insignificant for both outcomes (Table 2). Also, wide confidence intervals for the ADHD-RS-IV and SDQ-DAN scores indicated no significant differences between groups over time (Additional files 3, 4). No adverse events were reported by the participants.

**Table 1:** ADHD symptoms and functionality (SDQ-DAN) over time

Outcome measure	Time/month	TEAMS group			Control group			Between group	95% confidence interval (CI)
		N	Mean	SD	N	Mean	SD	Mean diff.	Difference—CI^a^
ADHD-RS-IV	Pre	33	69.15	12.61	26	74.62	10.86	− 5.46	− 11.70 to 0.77
	Post	32	65.75	12.91	7	63.57	12.04	2.18	− 8.62 to 12.98
	1	33	65.67	12.79	4	63.75	10.31	1.917	− 11.63 to 15.46
	3	32	65.25	13.04	6	59.67	12.21	5.583	− 6.08 to 17.25
	6	33	63.33	12.82	7	59.43	10.95	5.218	− 6.66 to 14.47
SDQ-DAN	Pre	33	46.91	5.94	23	48.87	5.08	− 1.96	− 5.01 to 1.09
	Post	30	44.53	5.44	7	45.86	3.24	− 1.32	− 5.69 to 3.05
	1	27	45.59	4.53	6	45.00	2.97	0.59	− 3.38 to 4.57
	3	26	44.96	4.36	7	47.14	3.81	− 2.18	− 5.88 to 1.52
	6	32	44.53	4.48	8	47.38	3.78	− 2.84	− 6.33 to 0.64

Means, standard deviations, mean differences and confidence intervals (CI) at baseline, posttreatment, and 1, 3, and 6 months follow up by group

^a^Independent samples T test

**Table 2:** Repeated measures GLM procedure of the effect over time and between groups (p values)

Group	Intervention group (time)	Intervention group (8 weeks*group)	Intervention group (time*group)
ADHD-RS-IV	0.020	0.110	0.524
SDQ-DAN score	0.166	0.896	0.608

Independent samples test

We conducted a post hoc trial sequential analysis (TSA) on the primary outcome ADHD symptoms at 8 weeks [18–23] (Additional file 5). The required information size was 130 participants. The cumulated Z-curve (blue curve) did not cross the trial sequential monitoring boundaries for benefits or harms (red inner sloping lines) or the trial sequential monitoring boundaries for futility (red outward sloping lines), implying that there was a risk of random errors. In accordance, the trial sequential analysis-adjusted confidence interval is − 19.3 to 23.7.

### Discussion

No significant improvements on ADHD symptoms or functionality were seen over time in the TEAMS intervention compared with the standard treatment. There was substantial dropout in the standard treatment group, with 19 out of 26 participants (73%) withdrawing during the intervention period. This was mainly because parents in the standard treatment group wanted their children to initiate pharmacological treatment. The parents in the TEAMS group seldom raised this request.

The Danish health authorities recommend non-pharmacological treatments for preschool children with ADHD [17]. We chose to exclude medicated children in the trial to accurately measure the effects of a non-pharmacological intervention only. Many parents held that medication would make for a smoother transition from kindergarten to primary school, and dampen future academic, social, familial, or personal conflicts. However, methylphenidate and other stimulant medication may only have little, if any, clinically important effects on ADHD symptoms [7]. Like other stimulants, methylphenidate is also connected with a number of adverse events [8].

This trial has some strengths. We published a protocol beforehand, the outcome assessors were blinded to treatment allocations at baseline assessment, and we only used two outcome measures, which may have protected for multiplicity issues due to random errors [24, 25]. Publication bias is a prominent problem in intervention research [26, 27], and it is important that null findings like these are reported in a transparent fashion. The families and their networks were also offered a high level of psychoeducation during treatment, which is considered a good treatment strategy for ADHD in Denmark [17]. Large-scale randomised clinical trials are needed to determine the true clinical utility of TEAMS training.

## Limitations

The required sample size of 90 participants was not reached, because there were too few referrals in the target age group during the time of enrolment. Thus, the trial is underpowered to detect a true difference (i.e. at risk of type II errors). We also experienced dropouts during the initial baseline assessment on sociodemographic factors. The large and uneven attrition rates likely confounded the results as well [28]. We planned to conduct intention-to-treat analyses and multiple imputation for missing follow-up data (> 5%), but decided not to due to the substantial dropout rates. There is no easy fix to attrition rates greater than 20%, and it is unlikely that adjustment strategies would have been able to save the data [28, 29].

## Additional files

**Additional file 1.** Baseline characteristics (sociodemographic and clinical variables) for the TEAMS- and control group.

**Additional file 2.** ANCOVA model of the effect after eight weeks and between-groups^a^.

**Additional file 3.** Box plot of differences between groups over time for ADHD scores. Group 1 = TEAMS and Group 0 = Control.

**Additional file 4.** Box plot of differences between groups over time for SDQ scores. Group 1 = TEAMS and Group 0 = Control.

**Additional file 5.** Trial Sequential Analysis on ADHD symptoms. MRDIF = Minimal relevant difference.

## Abbreviations

AHHD: attention deficit hyperactivity disorder
TEAMS: the training, executive, attention, and motor skills program
ADHD-RS IV: the ADHD rating scale-IV
SDQ-DAN: the strengths and difficulties questionnaire—Denmark
GLM: general linear model
ANCOVA: analysis of covariance model
