# Supplementary material for: Training, executive, attention and motor skills (TEAMS) training versus standard treatment for preschool children with attention deficit hyperactivity disorder: a randomised clinical trial
Source: BMC Res Notes. 2018 Jun 8;11:366. doi: 10.1186/s13104-018-3478-3 (PMC5994071; doi:10.1186/s13104-018-3478-3)
Supplement: Supplementary file 1 — Additional file 1. Baseline characteristics (sociodemographic and clinical variables) for the TEAMS- and control group. [file 13104_2018_3478_MOESM1_ESM.docx]

**Additional file 1**

|  | TEAMS | Control | P value |
| --- | --- | --- | --- |
| Sociodemographic variables |  |  |  |
| Age (years) | 5.69; SD 0.97 (n = 27) | 5.65; SD 1.00 (n = 16) | 0.884^a^ |
| Sex (males %) | 84.9 (n = 33) | 92.3 (n = 26) | 0.379^b^ |
| Clinical variables |  |  |  |
| Baseline ADHD score (mean ±SD) | 69.15; SD 12.61 (n=33) | 74.62; SD 10.86 (n=26) | 0.079^c^ |
| Baseline SDQ score (mean±SD) | 46.91; SD 5.94 (n=33) | 48.87; SD 5.08 (n=23) | 0.191^c^ |

Baseline characteristics (sociodemographic and clinical variables) for the TEAMS -and control group.

# ^a^ Independent Samples T-Test

# ^b^ Chi-Squares Test

^c^ Independent Samples T-Test
