# Supplementary material for: Training, executive, attention and motor skills (TEAMS) training versus standard treatment for preschool children with attention deficit hyperactivity disorder: a randomised clinical trial
Source: BMC Res Notes. 2018 Jun 8;11:366. doi: 10.1186/s13104-018-3478-3 (PMC5994071; doi:10.1186/s13104-018-3478-3)
Supplement: Supplementary file 2 — Additional file 2. ANCOVA model of the effect after eight weeks and between-groupsa. [file 13104_2018_3478_MOESM2_ESM.docx]

**Additional file 2**

ANCOVA model of the effect after eight weeks and between groups.^a^

| Group | Mean | 95 % CI | Mean difference | Standard error | P-value |
| --- | --- | --- | --- | --- | --- |
| ADHD score: |  |  |  |  |  |
| Control | 62.78 | 59.24 – 66.31 | -3.15 | 0.815 | 0.111 |
| TEAMS | 65.92 | 64.27 – 67.58 |  |  |  |
| SDQ score: |  |  |  |  |  |
| Control | 45.06 | 42.12 – 48.01 | 0.35 | 0.697 | 0.831 |
| TEAMS | 44.72 | 43.30 – 46.14 |  |  |  |

^a^ ANCOVA ProAddicedure
