# Supplementary figures and images for: Training, executive, attention and motor skills (TEAMS) training versus standard treatment for preschool children with attention deficit hyperactivity disorder: a randomised clinical trial
Source: BMC Res Notes. 2018 Jun 8;11:366. doi: 10.1186/s13104-018-3478-3 (PMC5994071; doi:10.1186/s13104-018-3478-3)

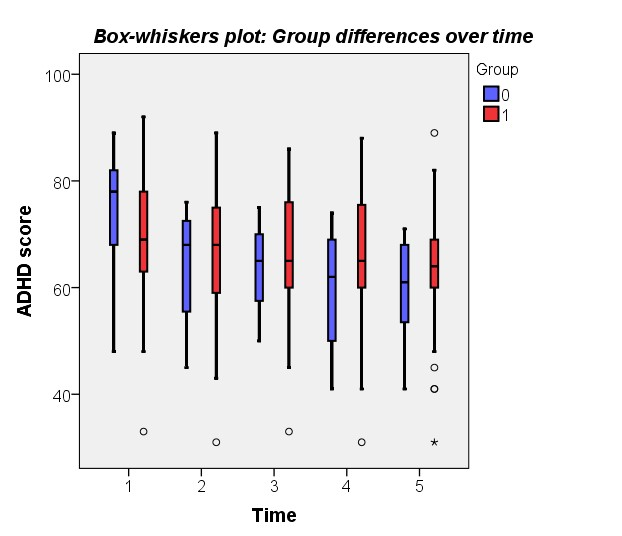

Supplement: Supplementary file 3 — Additional file 3. Box plot of differences between groups over time for ADHD scores. Group 1 = TEAMS and Group 0 = Control. [file 13104_2018_3478_MOESM3_ESM.tif]

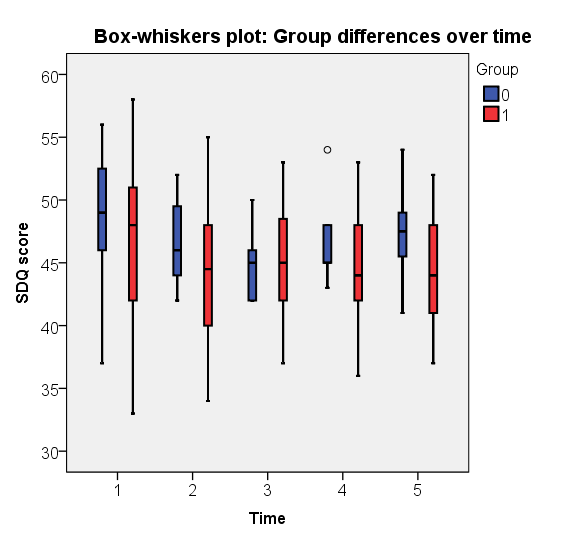

Supplement: Supplementary file 4 — Additional file 4. Box plot of differences between groups over time for SDQ scores. Group 1 = TEAMS and Group 0 = Control. [file 13104_2018_3478_MOESM4_ESM.tif]

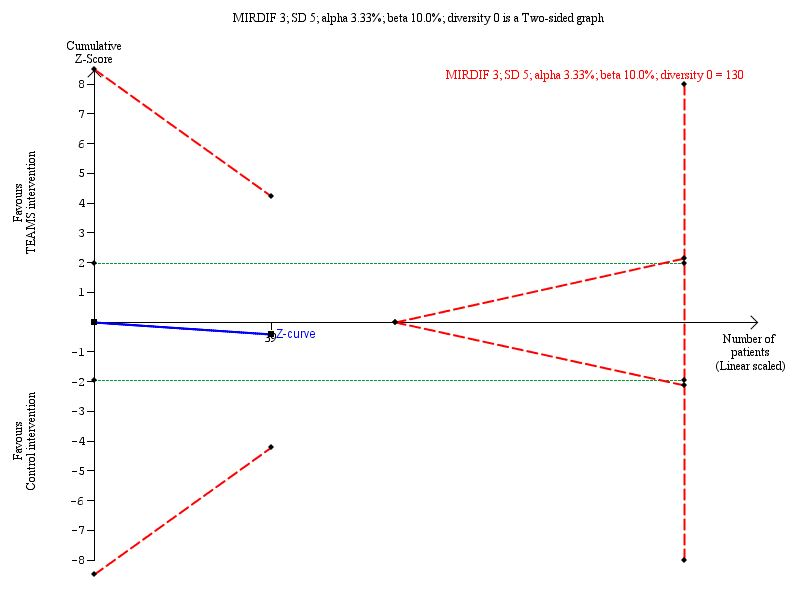

Supplement: Supplementary file 5 — Additional file 5. Trial Sequential Analysis on ADHD symptoms. MRDIF = Minimal relevant difference. [file 13104_2018_3478_MOESM5_ESM.tif]
